# Reducing Antimicrobial Use by Implementing Evidence-Based, Management-Related Prevention Strategies in Dairy Cows in Switzerland

**DOI:** 10.3389/fvets.2020.611682

**Published:** 2021-01-18

**Authors:** Manuela Gerber, Salome Dürr, Michèle Bodmer

**Affiliations:** ^1^Clinic for Ruminants, Vetsuisse-Faculty, University of Berne, Berne, Switzerland; ^2^Veterinary Public Health Institute, Vetsuisse Faculty, University of Bern, Bern, Switzerland

**Keywords:** dairy cattle, animal health, antimicrobial usage reduction, farmer, implementation of control strategies

## Abstract

The aim of this study was to reduce antimicrobial use (AMU) on dairy farms that implemented evidence-based management strategies. The study not only examined whether these strategies led to a reduction in AMU in practice, but also examined the influence of the level of their implementation on AMU. For data analysis, practice software recordings of the farm veterinarians were used. The AMU data of 50 farms with prevention strategies applied (intervention group, IG) over 3 years (2017–2019) and of 74 farms without prevention strategies (control group, CG) over 2 years (2018–2019) were analyzed. Project participation was supported with 500 Swiss francs (~545 USD) per farmer per year. The AMU was compared between the IG and CG using the treatment incidence. In December 2017/January 2018, the farmers of the IG had chosen at least one of the proposed 17 prevention strategies from one of three sectors, i.e., udder health, uterine health and/or replacement calf health. The prevention strategies, were developed in a standard operating procedure protocol and were discussed in detail with the farmers before the implementation. Forty-eight farms chose at least one udder strategy, 10 farms at least one uterine strategy and 37 farms at least one calf strategy. By choosing an udder health strategy or a uterine health strategy, the corresponding systemically administered AMU could be significantly reduced (*p* < 0.04) in the IG compared with the CG. In addition, udder strategies that were well-implemented led to a significant reduction (*p* = 0.05) of intramammary “highest priority critically important antimicrobials (HPCIA)” (quinolones, cephalosporins 3rd and higher generation, macrolides and ketolides, glycopeptides, and polymyxins). The level of implementation was significantly lower in 2019 compared to 2018 (*p* < 0.05, Fisher's exact test). No significant reduction in AMU could be achieved for the calf sector. A reduction of AMU in dairy farms is possible by implementing evidence-based management-related prevention strategies. The level of implementation has only an influence on the consumption of HPCIA. The reduction of AMU in practice by means of evidence-based measures requires supportive human resources instead of financial support, because financial support for farmers seems not to motivate them sufficiently.

## Introduction

The development of antimicrobial resistances in bacteria leads to major problems in both human and veterinary medicine. The current efforts of various institutions such as governments or the World Health Organization aim at reducing antimicrobial use (AMU) toward a targeted, reasonable and evidence-based AMU. Evidence-based means adherence to a procedure, in this case the treatment with antimicrobials (AMs), which is based on the best and most recent scientific knowledge.

The sales of AMs in veterinary medicine in Switzerland decreased from 76 mg/PCU (population correction unit: 1 PCU = 1 kg farm animal) in 2009 to 38 mg/PCU in 2019 (1). This reduction was mainly due to lower sales of drug premixes. In comparison with 30 EU countries, Switzerland ranks in 2017 the 21st among these countries, regarding AMU. However, Switzerland ranked first for highest intramammary AM drug user and second for intrauterine AM-containing preparations (2). Due to the fact that these two categories are used almost exclusively for cows and there are many more dairy cows [522,723 in 2018 (3)] than female, adult suckler cows [14,034 cows in 2018 (4)] in Switzerland, it can be assumed that this consumption is mainly attributable to dairy cattle.

On dairy farms, three common areas of AMU are udder health, uterine health and replacement calf health. Udder infection (mastitis) is one of the most common diseases in dairy cows. In most cases mastitis is caused by a bacterial infection. Cows with mastitis often need to be treated with AMs due to animal welfare reasons (5). Due to the high costs (treatment costs, decreased milk yield, milk withdrawal, premature culling), it is often preferable to prevent and control mastitis at farm level rather than to treat affected animals (6, 7). The incidence of mastitis can be reduced by controlling various factors. One of the most important factors is hygiene. Firstly, poor hygiene of the bedding in the lying area, caused by poor care of the bedding or heavily soiled claws and legs, increase bacterial load and thus the risk of mastitis (8–11). Secondly, metabolic diseases such as hypocalcemia (occurring mainly in the transition period), which cause recumbency, increase the risk of mastitis due to the long exposure of the teats with the bedding, contaminated with bacteria, and due to a weakening of the teat sphincter muscle (12). The immune system is also significantly weakened in metabolic diseases such as ketosis, so that the risk of mastitis increases (13, 14). Thirdly, good hygiene during milking, as well as correct post-milking teat-dipping, contributes to preventing bacteria enter the teat-canal during milking and mastitis being transmitted between cows (15, 16). The identification of the pathogen causing the mastitis is not only important in choosing of the active ingredient, but also for defining the optimal moment to start the therapy. In order to improve udder health, it is important to treat existing mastitis caused by contagious pathogens as quickly and efficiently as possible to keep the risk of transmission low (17).

Dairy cows developed uterine disease are often treated with AM to prevent a decrease in milk yield and poor fertility and reduce suffering by combining AM and pain medication (18, 19). However, prevention of postpartum uterine diseases should be preferred to treatment (20). Uterine health and fertility can be significantly improved and AMU reduced (21–24) by avoiding metabolic diseases such as hypocalcemia or ketosis, occurring mainly in the transition period, by also improving the overall immunity, health and environmental hygiene around calving and in obstetrics. Bacterial infections of the uterus (endometritis or metritis) may lead to decreased reproductive performance (25–29) and are therefore often treated with AMs (30). Since a retained placenta is one of the major predisposing factors for uterine infection, it is also often treated with AMs, although it is not an infectious disease *per se* (31).

In the calf sector (including veal calves and replacement calves) most AMs are prescribed to treat respiratory and gastrointestinal diseases (30, 32). The problem of AM resistances is also a major issue in calf disease (33–36). In order to improve the situation and to be able to reduce AMs while maintaining good animal health, calf management is an important factor together with the farmers' mindset (37). In addition to the type of housing and group size, the colostrum supply and administration of trace elements, for example, are management elements that influence the health and thus AMU of calves (38–41). Many treatments with AMs in calves are performed without previously consulting a veterinarian (42, 43) which may lead to untargeted or even unnecessary use of AM.

Of course, the veterinarian also has an important role when it comes to AMU on the farms. In Switzerland, farmers can only obtain AMs through their veterinarian. Only if the farmers have a defined contract with a veterinarian is it allowed to sell AMs to the farmers for future use, so that the farmers can initiate treatments of affected animals themselves (44). However, dispensing stockpiled medication is prohibited as a preventive use of AMs such as systematic drying- off in the herd (blanket dry cow treatment). The farmer must provide some evidence (e.g., cell count measurement of the cow or result of a milk analysis) in order for the veterinarian to allow the dispensing of AMs. It is completely forbidden to dispense HPCIAs for potential future treatments. Farmers can only sign this contract with one veterinarian at a time. The prescription of AMs by veterinarians is influenced by various factors, for example the knowledge and experience of the veterinarian (45–48). There is hardly any research on this topic specifically for Switzerland, although Carmo et al. showed a similar tendency (49).

Although there are many studies investigating management practices to reduce the effect of AMU, practicing veterinarians or farmers experience difficulties in their implementation. This is related to the fact that the research is often performed under study conditions with the greatest caution and effort (e.g., supervision and monitoring) provided for the entire study group. In practice, however, implementing new features is not always as easy as it seems at first sight. The translation of complex knowledge from research into practice is also described by De Vliegher et al. as one of the key points in the control of mastitis (17). In addition, personality, own experiences, motivators and knowledge of a person, in our case the farmers, influences greatly a change in behavior (41). This rationale has been summarized and described several times in different theories or behavioral models (50, 51). In the study presented here, we investigated for the first time the implementation level of management changes as a factor influencing AM reduction on Swiss dairy farms.

The objective of this study was to reduce AMs by implementing a selection of evidence-based management practices, and at the same time maintain good animal health on the farm with a maximum deterioration of health indices by 5%. We hypothesized that dairy farmers who implemented management changes will achieve a greater reduction in AMU than farmers who did not implement changes. Furthermore, we hypothesized that the higher the level of implementation of the prevention measures, the greater the reduction in AMU.

## Materials and Methods

### Study Population

This study evaluates data from a convenience sample. In total 138 dairy farmers participating in a voluntary, regional AM reduction project were included in this study. Sixty-four farmers chose to participate in the intervention group (IG) from January 2017 to December 2019 on a voluntary basis. In parallel, the remaining 74 dairy farmers served as a control group (CG) from January 2018 to December 2019.

Farmers of the IG were recruited during 2017. Veterinarians of the IG farmers collected data on AMU and farmers collected animal health data during 2017, serving as a reference before the application of an intervention. The interventions were then implemented from January 2018 to December 2019. The participating farmers all signed a declaration of consent that allowed the collection of treatment data of their animals from their veterinarian. In addition, all farmers signed a contract with the project management, in which they commit themselves implementing the selected strategies and for which they received financial compensation (see Antimicrobial Reduction Project).

In parallel, 74 dairy farmers (from the same region, producing under the same conditions and supervised by the same veterinarians as the farms in the IG) served as a control group (CG). The farms of the CG were recruited during 2019, with the aim of serving as intervention farms for a second phase of the project (2020–2024). In order to be able to compare the farms of the IG with those in the CG, the treatment data for the AMU and the animal health data of the CG farms were retrospectively collected from the veterinarians and the breeding associations for the years 2018 and 2019. As there were changes of veterinarians and some farms did not agree to analyze the data so far back, a retrospective analysis until 2017 was unfortunately not possible. Data of the CG were thus available for the years 2018 and 2019.

### Antimicrobial Reduction Project

Veterinarians of the IG farmers collected data on AMU and farmers collected animal health data during 2017, serving as a reference before applying an intervention. At the end of 2017, the intervention group selected at least one strategy of management changes from a set of 17 evidence-based prevention strategies (Appendix I) to be implemented in 2018 and 2019. The proposed strategies with preventive measures to be implemented on farms are based on current scientific knowledge and are considered efficient in terms of improving animal health or preventing disease. Prior to selecting the prevention strategies, each farm of the IG was visited by a veterinarian and an animal scientist and the problem areas of each farm were identified and discussed.

The set of preventive measures for selection consisted of strategies concerning the following three health areas: udder health, uterine health and calf health. All strategies aim to improve animal health through preventive measures (strategies 1–5 and 8–17) or to use the correct AMs in the sense of a targeted treatment (strategies 6 and 7). In brief, the strategies for controlling mastitis included systematic bacterial culturing of high somatic cell count and cows with clinical mastitis, improving overall barn hygiene in order to have an improved hygiene of the animals, improving the milking routine and hygiene during milking and preventing milk fever and ketosis. The strategies to prevent uterine disease included measures to improve hygiene around calving and prevention of milk fever and ketosis. Finally, the measures for preventing calf disease consisted of improving colostrum management, vaccination, improving barn hygiene, improving the feeding of calves and providing vitamins and trace elements.

Each farm was visited by the same veterinarian (MG) and by one of three animal scientists. This ensured that the procedure on the farms was as uniform as possible. Due to the individual analysis of the health situation on each farm, the discussion was, however, inevitably individual to the farm, despite the same procedure. A veterinarian (MB), in collaboration with animal scientists compiled the set of strategies using evidence-based knowledge. Strategies for udder health, uterine health and calf health were defined and elaborated and recorded in specific protocols. Based on these protocols, the farmer who chose the corresponding strategy was able to implement the strategy step by step.

These consultants discussed the current situation on the farms with the farmers, inspected the treatment journals and identified the problem areas on the farms. Afterwards, different prevention strategies were presented to the farmers in detail, of which they should choose at least one. The choice of strategy was then left exclusively to the farmers, and no recommendation was made by the consultants regarding as to which strategies would be preferable for their farms situation. The practical implementation was discussed with the veterinarian and the animal scientist at the beginning of 2018 and open questions could be clarified.

Participation in the AM reduction project was remunerated for farmers with 500.- Swiss Francs (~548.00 US Dollars) per farm per year. In addition, the participants of the IG who have chosen an udder strategy had their costs reimbursed for the bacteriological milk analyses and resistance testing by the project. For farmers who have chosen the colostrum management strategy, a colostrometer was provided free of charge.

### Data Collection

#### Antimicrobial Use

Farmers were recruited as study participants. The participating farmers signed a written agreement allowing access to the data recorded by their veterinarians. The farm veterinarians provided treatment data on the participating farms. On request, the veterinarians extracted the documented and invoiced medication used or dispensed during the study period from their practice software. Veterinarians are not obliged to keep this documentation, but they need it for invoicing their services to their customers, the farmers. As mentioned in the introduction, in Switzerland, only veterinarians who have a contract with the farmers for the supply of veterinary medicinal products are allowed to supply AMs without clinical consultation (44). For each Swiss farmer it is only possible to conclude such a contract with one veterinarian. The treatment and supply data collected by the farm veterinarian, with whom the farmer had a contract for supplying AMs, was chosen for each farm in our project.

During the data cleaning process, all AM treatments in dairy cows or replacement calves and heifers with the corresponding indication or diagnosis were extracted from the dataset received by the veterinarians. At the same time, the AM treatments in these datasets were classified into one of the following four categories: “Udder Health,” “Uterine Health,” “Calf Health,” and “Other” (e.g., claw health or digestive disorders, or drugs that were dispensed for future use without a clear indication or diagnosis) (Appendix II).

To classify antimicrobial agents according to their importance, the list from the World Health Organization (WHO) was used. In this list, fluoroquinolones and other quinolones, 3rd and 4th generation cephalosporins, macrolides and ketolides and glycopeptides (not approved for use in veterinary medicine) are classified as highest priority critically important antimicrobials (HPCIA). Alternatively, the list of the OIE (Office International des Epizooties) or the EMA (European Medicines Agency) were consulted, which focus more on veterinary medicine. However, Swiss legislation defines 3rd and 4th generation cephalosporins, macrolides and fluoroquinolones as antimicrobial agents that may not be supplied as stockpiled medication. This legal obligation has led the authors not to choose the OIE or EMA list, as it is less suitable for the Swiss legal situation.

#### Animal Health Data

The breeding associations (Swissherdbook, Holstein Switzerland and Braunvieh Schweiz) of the farms provided individual somatic cell count to enable assessment of udder health of the participating farms. The mean of the monthly yield corrected somatic cell count (YCSCC) were used as a health indicator for the herd.

$$\begin{array}{l} YCSCC=\;\frac{\sum \left(amount\;of\;milk\;x\;cell\;count\right)}{\sum amount\;of\;milk} \end{array}$$

Based on the monthly means, the geometric mean value was then determined over 3 months in order to obtain a quarterly value.

Furthermore, annual fertility data was determined by the breeding associations using the “voluntary waiting period,” the “calving to conception interval,” and the “calving interval” and were made available for the study. The “voluntary waiting period” describes the time after calving until the first insemination. The “calving to conception interval” includes the time between calving and the successful insemination. The “calving interval” describes the number of days between two consecutive calvings.

In Switzerland there is no standardized system for recording cattle disease except for reportable diseases. The only way to gather information on disease prevalence or incidence, which are both very important as key performance indicators for cattle health, was the recordings of the digital treatment journal completed by farmers and was meant to serve as a database to calculate disease incidence in the present project. However, the review of data quality (2018) showed that data collection by farmers only lead to incomplete and therefore not evaluable data.

#### Control of Strategy Implementation

For each strategy, the level of implementation was described as “implemented,” “partially implemented,” and “not implemented.” The implementation level of the strategies related to milk quality (strategy 6, 7, 8), the number of milk sample results per farm was compared to the number of cows exceeding individual cell count of 150,000 cells per ml in the monthly milk recording. This enabled an estimation of whether the milk from cows that needed to be analyzed according to the chosen strategy (Supplementary Information) were actually sampled and analyzed, as a measurement for the level of the strategy implementation. For strategies that result in drug applications (strategy 13, 14 and parts of strategy 2 and 10) the sales data of the veterinarian in relation to the number of animals in the respective age group in the herds were used to evaluate the level of implementation. This is possible because in Switzerland, vaccines and drugs for injection (like selenium, vitamin D3 or iron) and AMs are only available from the veterinarian. For strategy 1, 9 and 16 a farm visit of the veterinarian (MG) was needed to start the implementation at all. This includes the strategies with the BCS determination of all cows in the herd as well as the climate measurements in the calf barn. Each of these visits with resulting recommendations was summarized in a written report from the veterinarian (MG) addressed to the farmers. The implementation level of the remaining strategies (strategy 3, 4, 5, 11, 12, 15, and 17 plus parts of strategy 2 and 10) was assessed on site at least once a year by the same observer (MG) using standardized evaluation sheets (Appendix III). If a farm selected several strategies in the same health sector, the best implemented strategy was used to categorize the evaluation for the entire health sector.

### Data Analysis

For the IG data from January 2017 to and including December 2019 was analyzed. For CG, the data was analyzed from January 2018 to and including December 2019.

The data cleaning, processing and calculation of treatment incidences was done with Excel (Microsoft, 2020 Version 16.36). Statistical analysis was performed with R (http://cran.r-project.org, Version 3.3.0, Boston, MA, USA).

#### Calculation of Treatment Incidence

Treatment incidence (TI) was used as a measure in order to quantify AMU and to be able to compare the values internationally (5, 32, 52–56). The DDDvet (=defined daily dose) and DCDvet (=defined course dose) values published by EMA [224954/2016 (57)] were used to calculate this treatment incidence. By using DDDvet and DCDvet for calculating the TI, the dosage recommendation for each active substance used per animal species and application route were considered. The DDDvet gives the daily dosage recommendation, and the DCDvet gives the dosage recommendation for the entire duration of therapy according to EMA guidelines.

A treatment incidence was calculated for each health category and each quarter (Jan-March, April-June, July–Sep, Oct–Dec for the years 2017 to 2019). TI were calculated for all AMs as well as for the HPCIAs alone. The TI are given in the following units: TI IMMlact = defined daily dose for animals (DDDvet) per cow per 1,000 days, TI IMMdry = defined course dose for animals (DCD vet) per cow per 1,000 days, TI IUute = DDDvet per cow per 1,000 days, TI SYSudd and TI SYSute = DDDvet per cow per 1,000 days, TI for calves = DDDvet per calf per 1,000 days. The calculation is based on a standard weight of 600 kg for cows and 80 kg for replacement calves (58, 59). The TI are calculated as follows:

$$\begin{array}{l} TI\;“IMMlact”=\frac{Amount\;of\;used\;udder\;injectors}{DDDvet\;[\text{per teat}]\;x\;number\;of\;days\;x\;number\;of\;animals}\times \;1000\\ TI\;“IMMdry”=\frac{Amount\;of\;used\;udder\;injectors}{DCDvet\;[\text{per udder}]\;x\;number\;of\;days\;x\;number\;of\;animals}\times \;1000\\ TI\;“SYSudd”\\ TI\;“SYSute'=\frac{Amount\;of\;active\;substance\;[\text{mg}]\;}{DDDvet\frac{[\text{mg}}{\text{kg}]}\;x\;number\;of\;days\;x\;number\;of\;animals\;x\;standard\;weight\;cow(600\text{kg})}\times \;1000\\ TI\;“Other”\\ TI\;“IUute”=\frac{Amount\;of\;used\;uterine\;tabs}{DDDvet\;[\text{intruterine product per animal}]\;x\;number\;of\;days\;x\;number\;of\;animals}\times \;1000\\ TI\;“CALFtot”=\frac{Amount\;of\;active\;substance\;[\text{mg}]\;}{DDDvet\frac{[\text{mg}}{\text{kg}]}x\;number\;of\;days\;x\;number\;of\;animals\;x\;standard\;weight\;calf\;(80\text{kg})}\times \;1000 \end{array}$$

The number of cows and calves per farm was recorded by the breeding associations quarterly. To calculate TI CALF the number of animals younger than 7 months were counted. For all the other TIs the number of cows, defined as animals with at least one calving, were counted. This data was calculated by the breeding associations for each quarter.

Treatment data of heifers was not included at all and was also not counted for the number of animals. The exact number of days at risk (number of days per quarter) was used for the calculation of each TI.

#### Statistical Analysis

For the data evaluation, the sample sizes per strategy are too small for an individual evaluation of each strategy. For this reason, all strategies were allocated to the three health sectors, i.e., udder health (strategies 1–8), uterine health (strategies 9–11) and calf health (strategies 12–17) and the following analyses were carried out at this level.

Firstly, a descriptive analysis of the data was carried out to compare the study populations of the IG and CG. Non-normally distributed continuous and categorical data of two groups were compared by the Wilcoxon-rank-sum test. Comparison of categorical data was done by the Chisquare test. Secondly, to compare AMU between IG and CG at the beginning of the intervention, the 5th quarter (January to March 2018) of both groups was used, as this is the earliest quarter for which data was collected from both groups. The distribution of the TI was visually explored by histogram and statistically by the Shapiro-Wilk test.

Two types of mixed effect zero-inflated nested models were applied. In the first type of models it was investigated whether strategy selection had any influence on the TI, by comparing the CG farms with the IG farms. The TI (from January 2018 to December 2019) of the farms from the IG, whose farmers have chosen at least one strategy from the sector under investigation, was compared with the farms from the CG. The TI was used as the outcome and the number of prevention strategies selected was used as an independent fixed effect. The second type of models was applied to investigate the influence of the level of intervention implementation on the TI. Here, the TI (January 2017–December 2019) of IG farms, whose farmers had chosen a prevention strategy and implemented it with different quality was compared with farms from the IG whose farmers had not chosen a strategy in this area. The independent fixed effect variable was therefore the level of implementation, while 0 stands for farmers not chosen a strategy, and 1–3 for farmers that have chosen a strategy with different levels of implementation (1 = implemented, 2 = partially implemented, 3 = not implemented).

For both model types, farms nested within quarters were used as random-effects in the models. The glmmTMB package in R for zero-inflated generalized linear mixed models was used for the analysis. In case of large enough sample size each model has been tested for region, breed and herd size by taking these variables into account as additional fixed effects. The model with the lowest AIC has been considered as the final model. For the zero-inflated parameter, depending on whether the zeros were equally distributed between the fixed effect levels, ziformula = 1 or ziformula = independent fixed effect was applied (60). Different distributions of the outcome variable (TI) were tested and the best fitting models, based on the Akaike information criterion (AIC), were used.

The level of significance was set at *p* ≤ 0.05.

## Results

Treatment and health data from 124 farms (IG *n* = 50, CG *n* = 74) provided by the veterinarian and the breeding association were evaluated. The data of 14 farms was not included at all because of following reasons: cancellation due to time constraints during the project period (*n* = 7), cancellation due to health reason of the farmer during the project period (*n* = 1), and no health data available from the veterinarian (*n* = 6). The last 6 months of the project period (July–December 2019) were not evaluated by two farmers, as one of them had quit the project in September 2019 and one had made massive management changes on the farm (merging of two herds in a new barn).

### Study Population

The different farm characteristics such as herd size, breed, milk yield, region, and number of different veterinarians per group are listed in Table 1.

**Table 1:** Description of farm characteristics of the intervention and control group.

	**Intervention group**	**Control group**
Number of farms	50	74
Average herd size		
-Number of cows with at least one calving	45.8 (SD 26.1)	37.0 (SD 21.9)
-Number of replacement calves (younger than 7 months)	15.9 (SD 9.7)	12.9 (SD 7.4)
Average milk yield (kg/year/cow)	8,542 (SD 88.3)	8,062 (SD 876.3)
**Cow breed**		
Dairy breed farms^*^	43 (86%)	45 (60.8%)
Dual purpose breed farms^**^	3 (6.0%)	14 (18.9%)
Number of farms with a combination of breeds	4 (8.0%)	15 (20.3%)
**Region of the farm**		
Valley region farms	20 (40%)	28 (37.9%)
Hill region farms	13 (26%	24 (32.4%)
Mountain region farms	17 (34%)	34 (29.7%)
**Number of different veterinarians**		
Number of different veterinarians/ veterinary practices	12	14
Number of study farms per veterinarian/ veterinary practices	min.-max.: 1–9 median: 3.5	min-.max.: 1–12 median: 5.5

* *Dairy breeds = Holstein Friesian, Red Holstein, Jersey, Brown Swiss*.

** *Dual purpose breeds = Simmental, Swiss Fleckvieh, Montbéliard, Braunvieh, Original Braunvieh*.

The breeds of the study population differed significantly between the IG and the CG (*p* < 0.05, Pearson's Chi-square test). The region distribution between the IG and CG do not differ significantly (*p* > 0.05, Pearson's Chi-square test). The herd size regarding adult cattle differed significantly between IG and CG (*p* < 0.05, Wilcox rank sum test). The average milk yield per cow per year per farm from 2017 to 2019 differed significantly between the two groups (*p* < 0.05, Wilcox rank sum test). The remaining farm characteristics do not differ significantly between the two groups.

### Choice of Prevention Strategies

In 48 out of 50 IG farms, farmers chose strategies to improve udder health 114 times. Strategies to improve uterine health were chosen 12 times by 10 different farmers. Thirty seven different farmers selected 64 strategies related to improving calf health.

Table 2 shows which strategy has been selected by how many farmers. The three most chosen strategies are strategy 6 (*n* = 29) and 8 (*n* = 32) out of the udder health sector and strategy 13 (*n* = 19) out of the calf health sector.

**Table 2:** Number of farmers who chose a strategy.

**Udder health**	**Topic**	**Number of farmers**
Strategy 1	Strengthening of the immune system	16
Strategy 2	Prevention of concomitant diseases (like milk fever)	11
Strategy 3	Hygiene of the lying surface	4
Strategy 4	Hygiene of the alleys	3
Strategy 5	Reduction of infections during milking	6
Strategy 6	Control plan for subclinical mastitis	29
Strategy 7	Treatment of clinical mastitis without fever	13
Strategy 8	Treatment for drying off	32
**Uterine health**		
Strategy 9	Strengthening of the immune system	7
Strategy 10	Prevention of concomitant diseases (like retained placenta)	4
Strategy 11	Reduction of microbial pressure at birth	1
**Calf health**		
Strategy 12	Colostrum management	11
Strategy 13	Administration of essential elements	19
Strategy 14	Vaccination	14
Strategy 15	Calf housing	7
Strategy 16	Calf keeping and stable climate	9
Strategy 17	Calf feeding	4

### Level of Implementation of Prevention Strategies

The level of implementation was significantly inferior in 2019 compared to 2018 (*p* < 0.05, Fisher's exact test). Table 3 shows the classification of the implementation level of health sector by each farmer.

**Table 3:** Number of farmers (*n* = 50) who implemented the prevention strategies in 2018 and 2019.

	**2018**			**2019**		
	***Implemented***	***Partially implemented***	***Not implemented***	***Implemented***	***Partially implemented***	***Not implemented***
Udder health (*n* = 48)	34 (70.8%)	6 (12.5%)	8 (16.7%)	33 (68.8%)	2 (4.1%)	13 (27.1%)
Uterine health (*n* = 10)	8 (80.0%)	2 (20.0%)	0 (0.0%)	8 (80.0%)	1 (10.0%)	1 (10.0%)
Calf health (*n* = 37)	25 (67.6.0%)	4 (10.8%)	8 (21.6%)	23 (62.2%)	3 (8.1%)	11 (29.7%)

*The level of implementation was divided into “implemented”, “partially implemented” and “not implemented”*.

### Use of Antimicrobials

The TI of the 5th quarter between the IG and CG did not differ significantly (*p* > 0.05, Wilcoxon rank sum test), except for the use of HPCIAs in the two subcategories SYSudd and SYSute (*p* < 0.05, Wilcoxon rank sum test), in which the use was higher in the IG. This indicated that at the beginning of the intervention, when we do not yet expect the intervention to influence the TI, the AMU is comparable between the two groups.

Table 4 shows the descriptive statistics of TI per health sector and (if applicable) per administration route. This table contains all farms of the respective group, independent of the number of strategies implemented and the level of implementation, respectively. When investigating those two effects [number of strategies implemented (model 1) and the level of implementation (model 2)], most of the TI categories did not change significantly (Table 5). For some models the region, breed and/or herd size resulted in an improvement of the model fit and were therefore included as fixed effects in the final models.

**Table 4:** Treatment incidence per group (median, interquartile range, 2.5th percentile, 97.5th percentile) of antimicrobials for the intervention group and the control group.

		**Treatment incidence 2017**				**Treatment incidence 2018**				**Treatment incidence 2019**			
		***Median***	***IQR***	***2.5th percentile***	***97.5th percentile***	***Median***	***IQR***	***2.5th percentile***	***97.5th percentile***	***Median***	***IQR***	***2.5th percentile***	***97.5th percentile***
IMMlact	IG	6.35	10.24	0.00	39.34	4.21	9.95	0.00	30.65	7.37	10.36	0.00	40.35
*DDD per cow per 1,000 days*	CG					5.95	10.70	0.00	37.04	6.21	10.17	0.00	32.09
IMMlact HPCIA	IG	0.00	0.65	0.00	10.00	0.00	0.00	0.00	5.88	0.00	0.57	0.00	7.22
*DDD per cow per 1,000 days*	CG					0.00	0.00	0.00	6.80	0.00	0.00	0.00	7.92
IMMdry	IG	0.32	1.38	0.00	4.68	0.34	1.19	0.00	3.47	0.50	1.46	0.00	4.50
*DCD per cow per 1,000 days*	CG					0.33	1.36	0.00	5.07	0.45	1.57	0.00	4.64
SYSudd	IG	0.48	1.88	0.00	9.24	0.55	1.97	0.00	9.26	0.85	1.92	0.00	6.47
*DDD per cow per 1,000 days*	CG					0.14	2.04	0.00	7.64	0.17	2.02	0.00	6.23
SYSudd HPCIA	IG	0.00	0.04	0.00	3.07	0.00	0.00	0.00	3.40	0.00	0.06	0.00	2.21
*DDD per cow per 1,000 days*	CG					0.00	0.00	0.00	2.91	0.00	0.00	0.00	1.64
SYSute	IG	0.00	0.47	0.00	1.51	0.00	0.47	0.00	2.84	0.00	0.24	0.00	3.03
*DDD per cow per 1,000 days*	CG					0.00	0.63	0.00	7.64	0.00	0.35	0.00	6.23
SYSute HPCIA	IG	0.00	0.00	0.00	0.01	0.00	0.00	0.00	0.59	0.00	0.00	0.00	0.00
*DDD per cow per 1,000 days*	CG					0.00	0.00	0.00	2.91	0.00	0.00	0.00	1.64
IUute	IG	0.82	2.82	0.00	5.23	0.73	1.67	0.00	5.23	0.67	2.91	0.00	8.27
*DDD per cow per 1,000 days*	CG					0.58	2.75	0.00	7.54	0.51	2.14	0.00	8.89
CALFtot	IG	5.25	18.32	0.00	73.23	1.76	14.1	0.00	112.03	0.74	7.09	0.00	64.47
*DDD per calf per 1,000 days*	CG					0.00	8.95	0.00	80.97	0.00	10.56	0.00	71.32
CALF tot HPCIA	IG	0.00	1.58	0.00	21.97	0.00	0.00	0.00	38.75	0.00	0.00	0.00	8.24
*DDD per calf per 1,000 days*	CG					0.00	0.00	0.00	6.91	0.00	0.00	0.00	6.79
Other	IG	2.18	5.35	0.00	26.12	2.46	4.70	0.00	22.55	2.03	5.21	0.00	25.00
*DDD per cow per 1,000 days*	CG					1.92	4.55	0.00	12.06	1.63	3.94	0.00	13.43
Other HPCIA	IG	0.00	0.00	0.00	6.53	0.00	0.00	0.00	6.81	0.00	0.21	0.00	3.39
*DDD per cow per 1,000 days*	CG					0.00	0.00	0.00	4.34	0.00	0.00	0.00	1.81

*DDD, defined daily dose; DCD, defined course dose; IG, intervention group; CG, control group; IMMlact, intramammary use during lactation; IMMdry, Intramammary use for dry-off; SYSudd, Systemic use with indication udder health; SYSute, systemic use with indication uterine health; IUute, intrauterine application; CALFtot, use for calves; HPCIA, Highest priority critically important antimicrobials; IQR, interquartile range*.

**Table 5:** Estimates (upper line) and *P*-values (lower line) of the conditional mixed effect regression models investigating the association between treatment incidence and amount of chosen prevention strategies (Model 1), and between treatment incidence and implementation level of the strategies (Model 2, 1 = implemented, 2 = partially implemented, 3 = not implemented).

**TI category**	***MODELS 1: Amount of chosen strategies***						***MODELS 2: Level of implementation***			
	**1**	**2**	**3**	**4**	**5**	**IFE**	**Implemented**	**Partially implemented**	**Not implemented**	**IFE**
**EFFECT OF UDDER HEALTH PREVENTION STRATEGIES**										
IMMlact	−0.21	0.03	−0.18	0.16	−0.17		0.07	0.03	−0.04	H^*^
	0.20	0.81	0.28	0.47	0.59		0.37	0.88	0.75	** <0.01**
IMMlact	2.46	0.04	0.73	3.16	0.70		**−0.27**	−0.85	0.10	H^*^
HPCIA	0.11	0.97	0.66	0.14	0.80		0.05^*^	0.27	0.64	** <0.01**
IMMdry	−0.25	−0.23	−0.14	0.13	−0.14	H^*^	0.02	**−0.51**	0.18	H^*^
	0.22	0.14	0.47	0.63	0.72	**0.04**	0.79	0.03^*^	0.19	** <0.01**
SYSudd	**−0.44**	−0.19	−0.15	0.05	0.17	H^*^,R^*^	0.04	−0.09	−0.13	H^*^
	0.04^*^	0.26	0.47	0.87	0.65	** <0.03**	0.71	0.70	0.51	**0.02**
SYS udd	−1.73	−0.65	1.26	1.42	−0.03	R^*^	0.10	0.17	**−0.87**	
HPCIA	0.20	0.53	0.33	0.42	0.99	**0.01**	0.57	0.72	0.03^*^	
OTH	−0.22	**0.47**	0.22	**0.78**	0.35	R^*^,H^*^	−0.04	0.18	−0.04	R^*^
	0.25	<0.01^*^	0.25	<0.01^*^	0.29	** <0.04**	0.68	0.42	0.80	** <0.01**
OTH	2.00	0.64	0.93	2.41	1.44	B^*^, R^*^	−0.27	−0.26	−0.11	R^*^
HPCIA	0.16	0.53	0.49	0.17	0.53	** <0.04**	0.11	0.52	0.76	**0.02**
**EFFECT OF UTERINE HEALTH PREVENTION STRATEGIES**										
IUute	−0.16	0.01				H^*^	−0.23	−0.55	0.44	H^*^
	0.45	0.98				** <0.01**	0.26	0.23	0.42	** <0.01**
SYSute	**−0.56**	0.25				H^*^	−0.03	−18.39	−0.15	H^*^
	0.02^*^	0.55				** <0.01**	0.89	0.96	0.87	** <0.01**
SYSute	1.12	−14.02				°	0.39	−23.85	−0.14	°
HPCIA	0.70	>0.99					0.91	1.00	>0.99	
OTH	−0.18	−0.47				R^*^	−0.12	−0.44	−0.46	R^*^
	0.38	0.25				**0.03**	0.52	0.28	0.48	**0.01**
OTH	2.28	−4.50				R^*^, H^*^	−0.14	0.18	−16.06	R^*^, B^*^
HPCIA	0.10	0.18				** <0.05**	0.77	0.96	0.99	** <0.01**
**EFFECT OF CALF HEALTH PREVENTION STRATEGIES**										
CALFtot	−0.26	−0.14	−0.28	0.88		R^*^	0.01	0.30	−0.34	
	0.26	0.60	0.28	0.17		**0.04**	0.97	0.44	0.15	
CALFtot	1.03	0.92	0.11	3.09		R^*^	0.17	−0.33	−0.04	H^*^
HPCIA	0.37	0.50	0.96	0.29		**0.02**	0.55	0.64	0.90	**0.04**
OTH	0.08	0.30	0.30	0.32		R^*^	0.02	−0.12	**−0.47**	R^*^
	0.59	0.12	0.26	0.44		** <0.01**	0.85	0.58	<0.01^*^	**0.01**
OTH	**2.45**	**−3.62**	2.52	3.90		R^*^	−0.18	0.44	−0.42	R^*^, B^*^
HPCIA	0.03^*^	0.02^*^	0.19	0.18		** <0.02**	0.28	0.26	0.15	** <0.02**

*The estimate represents the in- or decrease per 100 units of the respective treatment incidence. (Reference value: models 1: Control farms without intervention, models 2: Farms of the intervention group without strategy selection in this health sector). Significant additional fixed effects such as herd size, region and/or breed have been included on some models where they improved the model fit (lowest p-value indicated)*.

*TI, treatment incidence; IMMlact, intramammary use during lactation; IMMdry, Intramammary use for dry-off; SYSudd, Systemic use with indication udder health; SYSute, systemic use with indication uterine health; IUute, intrauterine application; CALFtot, total use of antimicrobials for replacement calves; OTH, antimicrobial use with other indications; HPCIA, Highest priority critically important antimicrobials; IFE, included fixed effects; H, herd size; B, breed; R, region*.

* *Indicates the p-value, which is interpreted as significant (p ≤ 0.05 = significant)*.

*Bold numbers = marked estimate, p-value and additional fixed effect, which are defined as significant*.

° *Inclusion of herdsize, breed and region as additional fixed effect was not possible for these models due to too small sample size in at least one category of each additional fixed effect*.

A statistically significant difference between the TI SYSudd of CG farms and farms with a chosen udder strategy was observed with IG using less AMs than CG farms (*p* = 0.04). Similarly, a significantly higher TI SYSute was observed in CG farms compared to IG farms with a chosen uterine strategy (*p* = 0.02). Furthermore, in the udder health sector the TI OTH increased for IG farms with two (*p* < 0.01) or four (*p* < 0.01) chosen udder health strategies, respectively. The choice of one or two calf health strategies had a significant impact on the TI OTH HPCIA. The TI OTH HPCIA increased for farms with a chosen calf health prevention strategy (*p* = 0.03). On the contrary the TI OTH HPCIA decreased for farms with two chosen calf health prevention strategies (*p* = 0.02). In addition, the models that studying the selection of calf strategies also showed that the region always had a significant influence on AMU. However, it was not always the same region that showed the same trend (increase or decrease).

It was shown that the high implementation level of udder strategies decreased the consumption of intramammary HPCIAs (*p* = 0.05). This is lost with a lower implementation level. The partial implementation of certain udder strategies had significantly decreased (*p* = 0.03) the TI for intramammary dry-off products. Interestingly, we found that farms with a chosen udder health strategy, could decrease TI SYSudd HPCIA only, if the strategies have not been implemented (*p* = 0.03). Similarly farms with chosen calf health strategies significantly (*p* < 0.01) reduced the AMU in the category OTH, only if the strategies were not implemented.

### Animal Health Data

Animal health indicators of the farms are presented in Table 6.

**Table 6:** Description of animal health data of the intervention and control group.

	**Intervention group**			**Control group**		
	**2017**	**2018**	**2019**	**2017**	**2018**	**2019**
**SCC (cells/ml)**						
-Mean -SD	132,109 73,564	132,577 75,837	132,060 67,665	–	128,158 69,784	130,932 75,322
**Calving interval (days)**						
-Mean -SD	405 29	408 24	407 24	–	403 25	403 26
**Voluntary waiting period (days)**						
-Mean -SD	82 13	81 13	82 13	–	85 15	89 18
**Calving to conception interval (days)**						
-Mean -SD	133 24	134 26	135 25	–	126 24	128 27

*SCC, somatic cell count; SD, standard deviation*.

Neither udder health (YCSCC) *(p* > 0.05, Kruskal Wallis test) nor fertility (calving interval, voluntary waiting period, calving to conception interval) (*p* > 0.05, Kruskal Wallis test) differed significantly during the study period. This applies to both the IG (2017–2019) and the CG (2018–2019).

## Discussion

This study investigated the effect of evidence-based prevention strategies on AMU in three health sectors, udder, uterus and replacement calves. The hypothesis that farmers which selected evidence-based management changes for disease prevention reduce AMU while maintaining animal health compared to farmers that did not selected management changes, can be partially accepted. Farmers that had chosen a prevention strategy in the udder or uterine area are able to significantly reduce the consumption of systemically applied AMs used for both sectors. However, there is no significant reduction in AMU in the TI of intrauterine use or the TI administered for calves. Likewise, the hypothesis that this AM reduction depends on the level of the implementation can be partly accepted. For three TI (IMMlact HPCIA, IMMdry and SYSudd HPCIA) it could be shown that the implementation level had a significant effect on the reduction of AMs. However, this effect could not be detected in the uterine and calf health sectors.

One reason why it was possible to reduce AMU, particularly systemically applied AMs in dairy cows, is that the number of acute and severe clinical cases (e.g., acute mastitis, acute metritis) could be decreased in IG farms, but the occurrence of less severe cases was not affected by the intervention and therefore the use of AM for local (intramammary and intrauterine) application did not change. This is based on the assumption that systemic AMs are more likely to be applied in severe cases than in mild cases (61, 62). It is encouraging to note that despite the observed reduction in systemic AMU, there was no increase in TI in locally applied preparations. In addition, the IUute, IMMlact, and IMMdry all show a tendency for a reduction in AMU when a strategy has been in place on the farm. In our study we found that the level of prevention strategy implementation mainly reduced the AMU of critically important antimicrobials. This confirms findings of a study investigating the impact of mastitis management on AMU in dairy herds, which shows that better mastitis management was associated with reduced usage of critically important AMs (55). Stevens et al. also formulated the hypothesis that poor management is being masked by the use of HPCIA (55). Fortunately, it can also be stated that AMU in the non-HPCIA classes, whilst it had not decreased it had not increased in any of the three areas. But as Table 5 shows, “Other HPCIA” is an exception. There, a significant increase in AMU was observed in connection with the selection of one calf health prevention strategy. However, it is also evident that there was a significant decrease in AMU when two strategies were selected. These two values are difficult to relate to a biologically plausible explanation. In this context we may have to consider that many of the farmers also kept veal calves in addition to the replacement calves and we cannot entirely discriminate if a treatment was given to a replacement calf or to a veal calf. However, it is known that purchased calves commingled on a farm for veal calf production are often more severely affected by diarrhea and pneumonia than replacement calves (63, 64).

Interestingly, also only partly implemented prevention strategies showed a significantly positive effect in reducing intramammary AMU for dry-cow treatment compared to non-implementation of udder strategies. This suggests that only certain parts of the prevention strategy are sufficient in terms of reducing AMU at dry-off; alternatively, this effect was driven by chance, due to the rather small sample size. Another possible explanation of seeing an effect of reducing AMU lies in the definition of partially implemented udder prevention strategies, which is based on the number of milk SCC analyses. In case a farmer decided to sample cows with an individual cell count threshold of higher than 150,000 cells/mL, less samples were taken than those using the suggested limit of 150,000 cells/mL. They are therefore categorized as a partially implemented prevention strategy, and at the same time potentially used less AM because of the lower chances of detecting subclinical mastitis cases. These treatments lead to the fact that the TI, in its entirety, could then not be reduced, although the implementation level according to the protocol was higher. This possible explanation shows once again the importance of the veterinarian as a farm consultant in monitoring and reviewing udder health, to name but one example (65). If drugs are used for these treatments without consulting the veterinarian, although this was strictly not advised in the strategies, farmers may lack the know-how about which bacteria should be treated at all and at which point in time the therapy is most successful. Better management is only one aspect for improving udder health, while quarter level factors such as pathogen specific risk factors (66) and also cow breed (17) make a difference. This fact leads to the circumstance that not all risk factors for a reduced animal health could be considered and changed by the prevention strategies applied to the farms and the reduction of AMs may therefore also be lower than expected. Additionally, it must be taken into account that a higher SCC threshold might be used to select cows for further investigation without a deterioration of the udder health at herd level. The threshold of 150,000 cells/ml was chosen taking Swiss legislation into account (67). The described decrease of the TI SYSudd HPCIA, when farmers have not implemented the chosen udder health strategy, is probably influenced by other factors than the proposed prevention strategies. These factors may include the veterinarian and his or her change of the therapy scheme or not investigated management changes, which improved animal health and therefore allowed a reduction of AMU.

AM reduction was not achieved in the replacement calf sector. This may have various reasons. AMU in replacement calves is assumed to be overestimated in this study, because not all calves' treatments could be clearly allocated to veal or replacement calves, respectively, due to missing information in the records. Whenever there was a doubt, a treatment was added to the replacement calves, except when the treatment explicitly mentioned that it was for a veal calf. As a result, the known high AMU in veal calves (68) is at least partially included in our treatment incidence of the comparatively small number of replacement calves. Furthermore, the selected management changes may have been implemented only in the replacement calves and explicitly not in veal calves that may have been present in close contact to the replacement calves on the same farm.

The results on strategy selection and implementation level allow conclusions why AMs could not be successfully reduced in all areas. On the one hand, it was found that not all strategies from one health sector were selected equally frequently. The fact that not every strategy is equally efficient in promoting animal health, has also to be considered. It is expected that if strategies with lower effect have been chosen, the reduction of AMU also is limited. On the other hand, we know from a previous questionnaire study in the autumn of 2018 that in many cases these farmers did not choose prevention strategies in their problematic area but rather according to their interest in gaining new knowledge (65). A possible explanation for this phenomenon is that the expected financial benefit is more motivating, or perhaps that they were perturbed by the effort required to solve a problem. Also, the lack of awareness of having a problem at all would be one possibility why farmers avoid implementing strategies in areas with existing animal health issues. Farm-specific and risk-based selection are however important because the determinants of AM treatment vary from one farm to another (69). Although a study of Swiss farmers showed that self-motivated management changes are better implemented, whether or not these are the most efficient strategies (70), investing into less problematic areas makes it even more difficult to reach an effect in AM reduction. Furthermore, the number of strategies per farm per health sector also varies substantially (range 1–5 prevention strategies). This fact may have two consequences. Firstly, we could assume that the more strategies chosen, the more AMs could be reduced. According to our data this is not the case. Secondly, it is also possible that the more strategies chosen, the greater the workload and the less careful the strategies were implemented, which was most probably an issue in our study. The systemic use of AMs for udder health can be taken as an example: with the implementation of a strategy, the use can be significantly reduced, but the more strategies chosen, the less the reduction tends to be. In addition to these explanations, the CG also had the opportunity to improve their management on their own initiative during the study. This would also explain the lack of difference in AMU between IG and CG, and the effect of the strategies themselves is underestimated. Another possibility why a reduction of AMs could only be observed in some health sectors is that the treatment strategies of the various farm veterinarians were very different and therefore the success of therapy was also very variable. For example, when AMs are saved for the treatment of acute cases and the alternative treatment was not successful, AMs saved in these cases must be used to treat chronic cases. Such a case cannot be ruled out by this study protocol without prescribed treatment protocols. For Swiss veterinarians there is a recommendation from the Swiss government: “Therapy guidelines for the use of antibiotics for veterinarians” (62). However, following this recommendation is not compulsory and all preparations approved in Switzerland may be used. Only the supply of AMs to the farmers for the application by the farmer itself is regulated (but additionally depends upon the label of the farm for example for the use of HPCIA).

Fortunately, there was no significant increase in TI in most sectors, with the exception of the TI of “Other AMs” of the udder health section. There, we observed a significant increase of the TI in farms with two or four strategies implemented, compared to no strategy implemented. We cannot identify a biologically meaningful explanation for this, but argue that the prevention strategies could hardly be the reason for this increase, because we most probably would have also detected an increase if three or five strategies are implemented.

The results presented here show that the implementation level decreased over time. This is an additional plausible explanation why no more significant reduction of AMs could be produced. Consistent implementation may fade out since there was no regular support during the study by veterinarians qualified in consultation and prevention. Similar findings were reported from study in Belgium in which worse implementation of recommended mastitis management criteria was observed in farms without veterinary support (55). In the current study, providing supervision was avoided to be able to investigate the effect of the strategies without regular support visits and consultation on the farm. However, for future planning of AM reduction initiatives, professional support by educated veterinarians should be considered as an imperative. The authors suspect that the financial support of the project participants provided little or no motivation in implementing the interventions sufficiently in the long term. Despite financial incentives and financial support for sample analysis, the implementation level decreased over time. In future projects, funding should rather be invested for close supervision and support, than for motivation of farmers. Another possibility would be to create an incentive system in which financial support is only provided after the implementation level has been checked. Previous findings show that both penalty and bonus systems are motivating for farmers (71). One example of a penalty system in Europe trying to motivate farmers to decrease AMU is the yellow card system in Denmark (72). A survey of Swiss farmers and veterinarians shows that a penalty system (for high AMU) is not desirable (73).

Within this study, it is not possible to draw conclusions on the efficiency of the individual strategies, since the sample size per strategy was too small. Through the continuation of the here described AM reduction project with a larger cohort, it may be possible to investigate the effect of single strategies in the coming years. As described earlier, an adapted management-changes selection based on a problem analysis, with professional and regular supervision, would surely be a helpful method of improving animal health (71). No statement can be made about the efficiency of individual management strategies, although different efficiency is expected (74). The two strategies to reduce the risk of ketosis and to reduce the risk of milk fever occur in both the uterine and the udder sector. This allows an interaction of the effect on all TI of these two sectors and there is no way to quantify the strength of this interaction. However, since the udder strategies were more often chosen than the uterine strategies, it can be assumed that reduced AM consumption in cases of uterine disease on a specific farm may have been influenced by the selection of udder strategies. Therefore, the effect of the uterine strategies might have been overestimated in farms with applications of both, udder and uterine strategies.

Selection bias due to voluntary participation in the study and an observer bias for the evaluation of the implementation level is likely to be present in the study. However, these biases are considered negligible. The selection bias is evenly distributed across the IG and the CG, because farmers of the CG are also participating voluntarily. It is possible that particularly committed, motivated and therefore “good” farmers participated in the study may have had an influence on the results. It is possible that the AMU of the participating farms was below average already before the project started and that the reduction of AMs could not be as high as it might have been in a broader study population. However, it must also be mentioned here that the implementation level is expected to be rather above average due to the high motivation of the farmers, and that a reduction of AMs could thus be achieved more easily. The observer bias is also estimated to be low, since the standardized assessment sheets used for the evaluation of the implementation quality are based on objective criteria and the evaluation was carried out strictly according to detailed scores, which exclude a subjective evaluation. It is assumed that the level of implementation on farms was systematically overestimated based on the classification of how the strategy per health sector per farm was implemented, even if several strategies were chosen. However, as this is the case in all the farms analyzed, there is no overestimation or underestimation among the intervention farms themselves.

It must also be assumed that the TI for SYSTlact, SYSTute, CALFtot, and OTHER is slightly underestimated, since not all long-acting preparations have a separate DDDvet value from the EMA. This means that the number of days with treatment is generally underestimated for these categories. This applies to both IG and CG, so no influence on the comparison of AMU is expected.

The CG differed significantly from the IG in breed, herd size and average milk yield. This fact could lead to the consequence that the reference AMU also differs between the two groups. With larger herds, the farmer is expected to be more professional and may therefore have better knowledge of dairy cow management. However, with a larger herd, it can also be argued that the number of treatments performed without veterinary consultation is higher and therefore inadequate or excessive AMU is possible. In the data set analyzed here, there is no evidence of a distortion of AMU with respect to herd size. Herds of up to 100 cows still count as suitable for family farming, therefore no major management differences in farming are expected between IG and CG. The larger proportion of dairy cows in the intervention group and the logically associated higher milk yield could mean a fundamentally higher AMU in the intervention group, especially regarding to the AMU as an indication of udder health. A statistical comparison of the treatment incidence of the first quarter in 2018 between the two groups showed a statistically significant difference only in the amount of HPCIAs in systemic application for udder and uterine health. However, this difference is interpreted to be negligible for the total AMU, because the total AMU in these two classes is not statistically significantly different and therefore only differs in the use of HPCIAs.

## Conclusions

A reduction of AMs in practice using evidence-based strategies is possible. In our study the TI of systemically administered AM for udder and uterine health could be reduced for farms where such a strategy was implemented. Furthermore, it could be shown that high implementation level of measures to improve udder health can significantly reduce the use of intramammary AMs. Maintaining the level of implementation of evidence-based prevention strategies in practice has been shown to be challenging. An appropriate evaluation of the value of financial vs. e.g., human resources should be considered in future project planning.

## Data Availability Statement

The original contributions presented in the study are included in the article/Supplementary Material, further inquiries can be directed to the corresponding author/s.

## Ethics Statement

As the written confirmation with the reference number (BASEC No.) Req-2018-00020 shows, according to the Cantonal Ethics Committee for Research, there is no obligation to obtain a license for this study. The reason for this is that the project does not fall under the Human Research Act. For this study, no ethically sensitive data were collected that were not approved.

## Author Contributions

MG, MB, and SD conceived and designed of the study and interpreted the study results. MG and MB performed the data collection. MG and SD performed the statistical analysis. MG wrote the first draft of the manuscript. All authors contributed to manuscript revision, read, and approved the submitted version.

## Conflict of Interest

The authors declare that the research was conducted in the absence of any commercial or financial relationships that could be construed as a potential conflict of interest.
