# Supplementary material for: Reducing Antimicrobial Use by Implementing Evidence-Based, Management-Related Prevention Strategies in Dairy Cows in Switzerland
Source: Front Vet Sci. 2021 Jan 18;7:611682. doi: 10.3389/fvets.2020.611682 (PMC7847904; doi:10.3389/fvets.2020.611682)
Supplement: Supplementary file 1 [file Table_1.docx]

*Appendix I Overview of all evidence-based management changes which could be chosen by the participating farmers and which should lead to a reduction of antimicrobials when applied as prevention strategies. (AM=antimicrobial)*

| **Udder health** | |
| --- | --- |
| *Prevention* | |
| Strategy 1 | Strengthening of the immune system   - Determination of the body condition score for all dairy cows (lactating and dry cows) by a veterinarian (MG), once a year for farms with > 30 cows and twice a year for farms with <30 cows - Graphic presentation and interpretation of the results (veterinarian and animal scientist) for the whole herd 🡪 written report of the results to the farmers - Making adjustments (suggested by the veterinarian and the animal scientist) in the feeding of the dairy cows if necessary |
| Strategy 2 | Prevention of concomitant diseases (like milk fever)   - Introducing a calcium-poor feeding adapted to the transition period. - Administer calcium boluses around birth, in problem cows (cows showing first symptoms of problems or cows already known to be susceptible to problems in this area (e.g. experience from last calving)) and all cows from the 3rd lactation onwards. - Administering vitamin D before birth to prepare the calcium metabolism optimally for birth and the preceding lactation period for problem cows and cows from 3rd lactation. |
| Strategy 3 | Hygiene of the lying surface   - Improve the hygiene of the lying surface by increasing daily cleaning (number adapted to the operation and already existing cleaning passages) |
| Strategy 4 | Hygiene of the alleys   - Improve the hygiene of the alleys by increasing the daily cleanings (number adapted to the operation and already existing cleaning passages) |
| Strategy 5 | Reduction of infections during milking   - Pre-milking in the teat cup - Teat cleaning before milking - Teat dipping at the end of milking (up to 30 seconds after cluster removal) - Semi-annual shearing or flaming of udders - Daily teat dipping from two weeks before calving - Milking order according to somatic cell count (SCC). Cows with SCC >150,000 at the end of milking. |
| *Prevention and treatment* | |
| Strategy 6 | Control plan for subclinical mastitis   - California Mastitis Test (CMT) for cows with SCC > 150,000 - Aseptic milk sample a quarter with a CMT >+ - Treatment according to the milk sample result after consultation with the veterinarian - susceptibility testing in case of one of the following germs and multiple occurrence: Klebsiellae, enterococci, coagulase-negative staphylococci, *S. aureus* - Monitoring of therapy success after *S. aureus* infection with milk analyses after withholding time - Elimination of therapy-resistant cows after three unsuccessful treatments for the same pathogen in the same lactation and failure with dry-off therapy |
| Strategy 7 | Treatment of clinical mastitis without fever   - Version 1   - Collect a milk sample in case of clinical mastitis   - Freeze milk sample   - Treatment with AM (standard therapy of the private veterinarian (routine treatment procedure as always applied by the veterinarian seeing a cow with clinical mastitis, not defined by the project management))   - If no response to treatment: analyse milk sample and adjust treatment - Version 2   - Collection and analysis of milk sample   - Milk the cow separately   - Regular temperature monitoring   - Treatment according to analysis and veterinary prescription |
| Strategy 8 | Treatment for drying off   - In all dairy cows with an average of SCC > 150,000 in the last three measurement: take and analyse a milk sample, treat with the appropriate AM - For the rest: CMT at least 5 days before drying off, CMT>+: take and analyse a milk sample, treat with the appropriate AM   Version 1   - The remaining cows (healthy) are dried off without teat sealant   Version 2   - The remaining cows (healthy) are dried off with teat sealant   Version 3   - The remaining cows (healthy) are dried off with teat sealant - Animals with a history of the following pathogens: *S. aureus, S. agalactiae, S. uberis* were treated after consultation with the private veterinarian   Version 4   - Select version 1, 2 or 3 plus additionally: - If cows have a milk yield of more than 20kg milk 10 days before drying off, they are fed low-nutrient feed or milk reducing products are administered - Sudden drying off |
| **Uterine health** | |
| Strategy 9 | Strengthening of the immune system   - Determination of the body condition score for all dairy cows (lactating and dry cows) - Making adjustments in the feeding of the dairy cows if necessary |
| Strategy 10 | Prevention of concomitant diseases (e.g. retention secundinarium)   - Introducing a calcium-poor feeding adapted to the transition period. - Administer calcium boluses around birth, in problem cows and all cows from the 3rd lactation onwards. - Administering vitamin D before birth to prepare the calcium metabolism optimally for birth and the preceding lactation period for problem cows and cows from 3rd lactation. |
| Strategy 11 | Reduction of microbial pressure at birth   - Clean and dry calving environment - Observance of hygiene in obstetrics - Place obstetric chains in disinfectant - Intervention only 2 hours after breaking the waters - Wash your hands - Wear gloves - Wash vagina clean with soap and water - Contact private veterinarian 12 hours after birth if the placenta has not been expelled   OR   - Temperature monitoring 2x daily - Multiple monitoring of the temperature development on the caving day - Daily temperature measurement from calving until 10 days after |
| **Calf health** | |
| Strategy 12 | Colostrum management   - Administer 4-6L of colostrum in the first 12 hours of the calf's life. - Administer colostrum by means of a probe if the calf does not drink on its own. - Measurement of the quality of colostrum using a colostrometer. - Replacing colostrum of insufficient quality with colostrum of the best quality from the frozen store. |
| Strategy 13 | Administration of essential elements   - Administering iron and/or selenium to the calf in the first weeks of life (alternative: selenium application to the mother) |
| Strategy 14 | Vaccination   - Vaccinating dams against diarrhoea or pneumonia (depending on the farm)   AND/OR   - Vaccinate calves in the first week of life with an intranasal pneumonia vaccine.   OR   - Vaccinate all calves with one vaccine (intranasally or subcutaneously depending on age) if more than two calves develop pneumonia at the same time. Furthermore, in this case, no AM group therapy via an automatic feeder may be used. |
| Strategy 15 | Calf housing   - Individual housing of calves in the first 3-4 weeks of life. - Cleaning of the calf housing system (e.g. igloo) using high pressure. - Disinfection of the calf housing system using a disinfectant adapted to the pathogen spectrum present. |
| Strategy 16 | Calf keeping and stable climate   - Measurement of various climate indicators in the calf barn (e.g. ammonia concentration, temperature and humidity) over several days at different times of the year. - If necessary, adjustments in the calf barn based on the measurements made. |
| Strategy 17 | Calf feeding  Version 1   - Heat milk (with radiator) to 40-42°C depending on the drinking technology - Per calf one bucket and nipple - Same drinking routine every day   Version 2   - In the first 3-4 weeks ad libitum drinkers with acidified milk if necessary - Clean daily and replace milk completely - Same drinking routine   Version 3   - Calf feeder: daily inspection and cleaning - Wash nipples daily - Same drinking routine |
