# Supplementary material for: Reducing Antimicrobial Use by Implementing Evidence-Based, Management-Related Prevention Strategies in Dairy Cows in Switzerland
Source: Front Vet Sci. 2021 Jan 18;7:611682. doi: 10.3389/fvets.2020.611682 (PMC7847904; doi:10.3389/fvets.2020.611682)
Supplement: Supplementary file 2 [file Table_2.docx]

*Appendix II Subdivision of health categories to calculate the incidence of treatment (IMMlat= intramammary use during lactation, IMMdry= Intramammary use for dry-off, SYSudd= Systemic use with indication udder health, SYSute= systemic use with indication uterine health, IUute= intrauterine application)*

| **Categories** | **Udder Health** | **Uterine Health** | **Calf Health**  **(CALFtot)** | **Other**  **(OTH)** |
| --- | --- | --- | --- | --- |
| *Subcategory 1* | Intramammary use during lactation (=IMMlact) | Systemic use with indication uterine health (=SYSute) |  |  |
| *Subcategory 2* | Intramammary use for dry-off (=IMMdry) | intrauterine application (=IUute) |  |  |
| *Subcategory 3* | Systemic use with indication udder health (=SYSudd) |  |  |  |
| *Subcategory 4* | Use with other application methods (e.g. topic) (=UDDoth) |  |  |  |

OTH = antimicrobials not occurring to one of the other three categories (e.g. claw health or digestive disorders, or drugs that were dispensed for future use without a clear indication or diagnosis)
