# Supplementary material for: Reducing Antimicrobial Use by Implementing Evidence-Based, Management-Related Prevention Strategies in Dairy Cows in Switzerland
Source: Front Vet Sci. 2021 Jan 18;7:611682. doi: 10.3389/fvets.2020.611682 (PMC7847904; doi:10.3389/fvets.2020.611682)
Supplement: Supplementary file 4 [file Data_Sheet_2.pdf]

Appendix III

Control visit for strategy 2 and 10

Farm ______________________________
Address ______________________________
Date ______________________________

1. Feeding

1.1 Low calcium feeding during transition phase

Low calcium feeding during transition phase ☐ ja ☐ nein

Unitl when ☐ > 2-3 weeks before calving
☐ 2-3 weeks before calving
☐ < 2 weeks before calving

Description winter ration: ______________________________
____
____

Description summer ration: ______________________________
____
____

1.2 Same feeding as in the following lactation

Start feeding with lactation ration during transition period:
- ☐ > 2-3 weeks before calving
- ☐ 2-3 weeks before calving
- ☐ < 2 weeks before calving
- ☐ no lactation ration during transition period

1.3 Feeding with concentrated feed

Start feeding with concentrate feed during transition period:
- ☐ > 2-3 weeks before calving
- ☐ 2-3 weeks before calving
- ☐ < 2 weeks before calving
- ☐ no concentrate feed at all during transition period

Amount of concentrate feed at calving

☐ < 2.5kg	☐ < 1/3 of the lactation ration of protein concentrate
☐ > 2.5kg	☐ > 1/3 of the lactation ration of protein concentrate

1.4 Adaption of mineral supply

Minerals during lactation: ______________________________
Which minerals? ______________________________
Minerals during transition period: ______________________________
Mineralbolus for transition period/at calving:______________________________

Control visit for strategy 3

Farm ____

Address ____

Date ____

1. Lactating cows

1.1 Dry litter

Kneel in the bedding for 5 seconds

☐ positive (= knees dirty and wet)

☐ negative (= knees dry and clean)

Hygiene Score cows

☐1 ☐2 ☐3 ☐4 ☐5

Source & Definition of each score: Reneau, J. K., Seykora, A. J., Heins, B. J., Endres, M. I., Farnsworth, R. J., & F. Bey, R. (2005). *Association between hygiene scores and somatic cell scores in dairy cattle. Journal of the American Veterinary Medical Association, 227(8), 1297–1301.*doi:10.2460/javma.2005.227.1297

Number of litter cleaning circles per day:______________________________

2. Dry cows

1.2 Dry litter

Kneel in the bedding for 5 seconds

☐ positive (= knees dirty and wet)

☐ negative (= knees dry and clean)

Hygiene Score cows

☐1 ☐2 ☐3 ☐4 ☐5

Source & Definition of each score: Reneau, J. K., Seykora, A. J., Heins, B. J., Endres, M. I., Farnsworth, R. J., & F. Bey, R. (2005). *Association between hygiene scores and somatic cell scores in dairy cattle. Journal of the American Veterinary Medical Association, 227(8), 1297–1301.*doi:10.2460/javma.2005.227.1297

Number of litter cleaning circles per day:______________________________

3. Chalk

Number of chalk supplementation per week:______________________

Amount used per chalk supplementation in total: ______________________

Product: ____

Grain size: ____

Control visit for strategy 5

Farm ____
Address ____
Date ____

- Pre-milking in the teat cup
  ☐ yes
  ☐ no

- Teat cleaning before milking
  ☐ yes
  ☐ no

- Teat dipping at the end of milking (up to 30 seconds after cluster removal)
  ☐ yes
  ☐ no

- Semi-annual shearing or flaming of udders
  ☐ yes
  ☐ no

- Daily teat dipping from two weeks before calving
  ☐ yes
  ☐ no

- Milking order according to somatic cell count (SCC). Cows with SCC >150’000 at the end of milking.
  ☐ yes
  ☐ no

Control visit for strategy 11

Farm ____
Address ____
Date ____

- Clean and dry calving environment
  ☐ yes
  ☐ no

- Observance of hygiene in obstetrics
  ☐ yes
  ☐ no

- Place obstetric chains in disinfectant
  ☐ yes
  ☐ no

- Intervention only 2 hours after the rupture of the bladder
  ☐ yes
  ☐ no

- Washing hands before obstetrics
  ☐ yes
  ☐ no

- Wear gloves
  ☐ yes
  ☐ no

- Wash vagina clean with soap and water before obstetrics
  ☐ yes
  ☐ no

- Contact private veterinarian 12 hours after birth if the placenta has not been expelled OR temperature monitoring 2x daily
  ☐ yes
  ☐ no

- Multiple monitoring of the temperature development on the calving day
  ☐ yes
  ☐ no

- Daily temperature measurement from calving until 10 days after
  ☐ yes
  ☐ no

Control visit for strategy 12

Farm ____

Address ____

Date ____

- Administer 4-6L of colostrum in the first 12 hours of the calf's life.
  ☐ yes
  ☐ no

- Administer colostrum by means of a probe if the calf does not drink on its own.
  ☐ yes
  ☐ no

- Measurement of the quality of colostrum using a colostrometer.
  ☐ yes
  ☐ no

- Replacing colostrum of insufficient quality with colostrum of the best quality from the frozen store.
  ☐ yes
  ☐ no

Control visit for strategy 15

Farm ______________________________
Address ______________________________
Date ______________________________

- Individual housing of calves in the first 3-4 weeks of life
  ☐ yes
  ☐ no

- Cleaning of the calf housing system (e.g. igloo) using high pressure
  ☐ yes
  ☐ no

- Disinfection of the calf housing system using a disinfectant adapted to the pathogen spectrum present
  ☐ yes
  ☐ no

Control visit for strategy 17

Farm ______________________________
Address ______________________________
Date ______________________________

VERSION 1

- Heat milk (with radiator) to 40-42°C depending on the drinking technology
  ☐ yes
  ☐ no

- Per calf one bucket and nipple
  ☐ yes
  ☐ no

- Same drinking routine every day
  ☐ yes
  ☐ no

VERSION 2

- In the first 3-4 weeks ad libitum drinkers with acidified milk if necessary
  ☐ yes
  ☐ no

- Clean daily and replace milk completely
  ☐ yes
  ☐ no

- Same drinking routine
  ☐ yes
  ☐ no

VERSION 3

- Calf feeder: daily inspection and cleaning
  ☐ yes
  ☐ no

- Wash nipples daily
  ☐ yes
  ☐ no
